# Supplementary material for: Investigating the relationship between Pfkelch13 mutations and response to artemisinin-based treatment for uncomplicated falciparum malaria: a protocol for a systematic review and individual patient data meta-analysis
Source: BMJ Open. 2025 Jul 8;15(7):e100251. doi: 10.1136/bmjopen-2025-100251 (PMC12243583; doi:10.1136/bmjopen-2025-100251)
Supplement: Supplementary file 2 [file bmjopen-2025-100251.reviewer_comments.pdf]

PEER REVIEW HISTORY

BMJ Open publishes all reviews undertaken for accepted manuscripts. Reviewers are asked to complete a checklist review form and are provided with free text boxes to elaborate on their assessment. These free text comments are reproduced below.

ARTICLE DETAILS

Title (Provisional)

A protocol for a systematic review and individual patient data meta-analysis investigating the relationship between Pfkelch13 mutations and response to artemisinin-based treatment for uncomplicated falciparum malaria.

Authors

van Wyk, Stephanie; Dahal, Prabin; Vouvoungui, Chistevy; Ayuen, Dhol S; Shokraneh, Farhad; Soma, Aboubakar; Watson, James A.; Guerin, Philippe; Barnes, Karen I

VERSION 1 - REVIEW

**Reviewer**	**1**
**Name**	**Mayor, Alfredo**
**Affiliation**	**Instituto de Salud Global Barcelona**
**Date**	**19-Mar-2025**
**COI**	**None**

The manuscript by Stephanie van Wyk et al entitled “A protocol for a systematic review and individual patient data meta-analysis investigating the relationship between

Pfkelch13 mutations and response to artemisinin-based treatment for uncomplicated falciparum malaria” describes a IPD meta-analysis protocol on the association between Pfkelch13 markers and artemisinin-based treatment response. The manuscript is well written, with a clear description of the planned analysis. Please, find below my comments that hopefully can contribute to increase clarity on the paper:

- The expected timelines of the analysis (data collection, analysis and dissemination) should be detailed in the paper (sorry if I missed this information).

- Table 1: Why reference 1 (and not other clinical studies) is included? Please describe what “WHO Low frequency” mean: is this a world-wide list of low frequency mutations described in reference 2?

- The analysis focusses on the response to artemisinin-based treatment. This implies symptomatic cases who require treatment, correct? Does this refer to fever? Should this information be a required (not only desirable), as it seems relevant for the analysis?

- In line with my previous comment, it seems to me that a molecular correction to distinguish reinfection and recrudescence is key to unambiguously describe treatment failures. I am surprised this information is only desirable (not essential), especially when it is described as an inclusion criterion.

- It would be very helpful to describe the rationale for key variables in the study, and detail how they are going to be used. For example, transmission intensity: I assume it is relevant as an indication of acquired immunity (as it is age), which can affect the ability of individuals to clear infections? How low/moderate/high is going to be defined (something is mentioned in the supplementary methods, but I think key variables should be described in the main manuscript). Is there any risk that these categories are not good enough for the analysis (if for example sites included in the analysis do not represent the whole spectrum of intensities, or if there is a strong collinearity between transmission and continent (high in Africa, low in Asia)? How is it going to be included in the analysis (the primary analysis described in line 319 does not seem to include this variable). I believe more details on these key variables (maybe described in a Table) would be very helpful.

- Line 215-6: "Studies evaluating treatment, such as mass drug administration, healthy volunteers, or prophylaxis or chemoprevention studies": The examples provided do not really evaluate treatment. Should the sentence this section (exclusion) be: Studies NOT evaluating treatment?

- How is the analysis going to take into account technical variability? For example, primary analysis include adjustment by parasitaemia, which is very much dependent on the microscopy quantification methodology (considered as a study metadata). How differences in the quantification can affect the analysis, and how they can be discarded or controlled for in the analysis? Similarly, the methodology to determine kelch13 mutations can have different performance and drive to different minor allele frequencies: Is this relevant? If so, how this will be taken into account? A better description on which methodological variabilities are important and how to treat them in the analysis would be very helpful.

- Who and where will the analysis be conducted? Analytical capacities (especially this kind of complex IPD analysis) is usually concentrated in the Global North, while the data to be analysed is originated in the Global South. How the team is planning to contribute to mitigate these capacity imbalances for the mid/long-term aim of ensuring that the means and capacity to actively participate in the analysis (and ideally conduct those analysis independently) is available where the health problem lies (subsidiarity principle)? Some discussion on this would be very helpful to build a responsible data sharing environment.

Reviewer 2

**Name**	**Assefa, Dawit**
**Affiliation**	**KNCV**
**Date**	**31-Mar-2025**
**COI**	**None**

The document is scientifically rigorous and well-organized, demonstrating a strong commitment to statistical integrity and ethical considerations. The analysis is thorough, and the conclusions are well-supported by the data.

While the introduction provides relevant background information, a clearer statement of the study's public health significance would enhance its impact. Additionally, a concise summary of the current knowledge gaps and the unique contribution of this analysis would help contextualize its importance.

Some statistical methods, particularly the selection of thresholds for phenotypic resistance, could benefit from further justification to ensure clarity for general readers who may not be familiar with prior studies. Similarly, a brief explanation of the rationale for using a normal mixture model to determine parasite clearance thresholds would strengthen the methodological transparency.

Specific points for clarification:

Line 25: Define Pfkelch13 for clarity.

Line 32: Provide a brief definition of PRISMA-IPD.

Line 39: Clarify the abbreviations IDDO/WWARN.

Lines 71-72: ACTs has already been defined earlier; consider removing the redundant definition.

Line 90: Specify what BTB/POZ refers to.

VERSION 1 - AUTHOR RESPONSE

Reviewer: 1
Prof. Alfredo Mayor, Instituto de Salud Global Barcelona, Centro de Investigação em Saúde de Manhiça
Comments to the Author:
The manuscript by Stephanie van Wyk et al entitled "A protocol for a systematic review and individual patient data meta-analysis investigating the relationship between Pfkelch13 mutations and response to artemisinin-based treatment for uncomplicated falciparum malaria" describes a IPD meta-analysis protocol on the association between Pfkelch13 markers and artemisinin-based treatment response. The manuscript is well written, with a clear description of the planned analysis. Please, find below my comments that hopefully can contribute to increase clarity on the paper:

Response: We thank the reviewer for taking the time to help us improve our manuscript. We greatly appreciate your insight and guidance.

- The expected timelines of the analysis (data collection, analysis and dissemination) should be detailed in the paper (sorry if I missed this information).

Response: We have updated the dates to the Methods section of the manuscript and Supplementary Materials file 2 Table 1. Lines 170-173 of the main manuscript have been added to the main text.

- Table 1: Why reference 1 is included? Please describe what "WHO Low frequency" mean: is this a world-wide list of low frequency mutations described in reference 2?

Response: The references have been corrected. We thank the reviewer for bringing this oversight to our attention.

The descriptions of these mutations have also been updated to clarify the intended meaning. Please see Lines 104-107: "Mutations previously identified by the WWARN K13 study group associated with slow parasite clearance. These mutations are classified as circulating at low frequency at the time of the analyses."

- The analysis focusses on the response to artemisinin-based treatment. This implies symptomatic cases who require treatment, correct? Does this refer to fever? Should this information be a required (not only desirable), as it seems relevant for the analysis?

Response: One objective of this IPD meta-analysis is to investigate the association between Pfkelch13 *mutations and parasite clearance dynamics, particularly delayed clearance and the prevalence of day-3 positivity, across diverse epidemiological settings. Thus, studies that only report parasite clearance parameters without following up with patients to assess their clinical and parasitological response may be included. The focus is on characterizing molecular and parasitological outcomes following ACT treatment rather than describing clinical presentation per se.*

While we acknowledge that fever (or a documented history of fever) at enrollment provides important clinical context and could influence parasite clearance metrics, mandating this variable may inadvertently exclude valuable datasets. Such exclusion would substantially limit geographic representation and temporal coverage, undermining the overarching aim of generating regionally and temporally relevant estimates of parasite clearance phenotypes. Nevertheless, whenever data on fever or body temperature are available, we will include these as per the WHO protocol for analysing Therapeutic Efficacy Studies.

- In line with my previous comment, it seems to me that a molecular correction to distinguish reinfection and recrudescence is key to unambiguously describe treatment failures. I am surprised this information is only desirable (not essential), especially when it is described as an inclusion criterion.

Response

We thank the reviewer for this thoughtful comment. We agree that molecular correction to distinguish reinfection from recrudescence is essential for accurately interpreting treatment failures. In our protocol, this variable is desirable for the literature screening stage to avoid prematurely excluding otherwise eligible studies that may not report PCR correction methods in the published manuscript. However, this information is routinely requested from all data contributors as part of our IPD acquisition process. As noted in Supplementary Materials S3, molecular correction methods are extracted or provided by the data contributor, reflecting that such methods are typically implemented in clinical studies, even if not explicitly reported in publications.

Therefore, while PCR correction is not required for a study to pass the initial screening, it is expected to be available from the source dataset and incorporated in our secondary outcomes analysis.

- It would be very helpful to describe the rationale for key variables in the study, and detail how they are going to be used. For example, transmission intensity: I assume it is relevant as an indication of acquired immunity (as it is age), which can affect the ability of individuals to clear infections? How low/moderate/high is going to be defined (something is mentioned in the supplementary methods, but I think key variables should be described in the main manuscript). Is there any risk that these categories are not good enough for the analysis (if for example sites included in the analysis do not represent the whole spectrum of intensities, or if there is a strong collinearity between transmission and continent (high in Africa, low in Asia)? How is it going to be included in the analysis (the primary analysis described in line 319 does not seem to include this variable). I believe more details on these key variables (maybe described in a Table) would be very helpful.

Response: We thank the reviewer for their helpful comments. We agree that transmission intensity and age-dependent immunity are critical modifiers of artemisinin responsiveness and parasite clearance dynamics. The revised manuscript clarifies these points [see the Supplementary materials S1 Section 1 Definition 2, ***Malaria transmission intensity*** *].*

Transmission intensity *is now defined in Supplementary Material S1, Section 1, Definition 2 and categorized as very low, low, moderate, or high based on* Plasmodium falciparum *prevalence and Annual Parasite Incidence (API), following WHO and Malaria Atlas Project criteria (Guerra* et al., *Malar J, 2007). These classifications are tailored to the location and year of patient enrolment.*

We agree with the reviewer that thresholds for delayed parasite clearance, such as the >5-hour PC½ commonly used in Southeast Asian contexts, may not be directly applicable in moderate or high transmission settings like sub-Saharan Africa, where baseline immunity often results in more rapid parasite clearance. The SAP addresses this issue in the "Thresholds for determining phenotypic resistance", on page 14 of the main manuscript. We note that this threshold will likely not generalize to African contexts. We describe our plan to

empirically estimate region-specific cutoffs using normal mixture modelling and 10-fold cross-validation, stratified by study site.

Furthermore, one of the stated objectives of this IPD meta-analysis is to compare parasite clearance dynamics and their association with Pfkelch13 *mutations across geographical regions, including comparisons between Asia and Africa, as well as stratification by transmission intensity. These planned analyses will ensure that differences in immunity and epidemiological context are rigorously accounted for in interpretation and surveillance recommendations.*

We have expanded the Methods section to include additional explanations for choosing phenotypic resistance thresholds. Please see lines 344-352 of the manuscript: The selection of thresholds for phenotypic resistance (for instance, Day 3 positivity >5% and a $PC_1/_2$ ≥5 hours), will be based on WHO guidelines and prior pooled analyses that identified these thresholds as indicative of delayed parasite clearance but will be re-evaluated in this analysis. A normal mixture model will be applied to the $PC_1/_2$ distribution to define subpopulations of fast- and slow-clearing parasites empirically. This statistical approach is commonly used when phenotypic data exhibit heterogeneity, allowing for a more robust estimation of cutoff points than arbitrary thresholds alone.

We thank the reviewer for this helpful suggestion regarding explaining the rationale and analytic role of key variables. Due to the journal's word count constraints in the main manuscript, we have addressed this request by consolidating all relevant information into a new table and accompanying explanatory text in the ***revised Supplementary Materials (now Supplementary Material S1 Tables S3)****. Definitions of key variables (e.g. age, baseline parasitaemia, transmission intensity), list the following information:*

- *Their biological and epidemiological relevance (e.g. proxies for acquired immunity),*
- *Data sources and classification criteria (e.g. WHO/MAP for transmission intensity),*
- *Their role in the statistical models (e.g. fixed effect covariates, stratifiers, or sensitivity variables),*
- *A note on potential limitations such as geographic collinearity and range representation.*

We have added cross-references to this new table in the main text where relevant, to guide readers to the Supplementary Materials.

- Line 215-6: "Studies evaluating treatment, such as mass drug administration, healthy volunteers, or prophylaxis or chemoprevention studies": The examples provided do not really evaluate treatment. Should the sentence this section (exclusion) be: Studies NOT evaluating treatment?

Response: We have amended the sentence to make the intended meaning clear. Please refer to lines 231: "● Studies that do not evaluate treatment of malaria, such as mass drug administration, healthy volunteers, or prophylaxis or chemoprevention studies….."

- How is the analysis going to take into account technical variability? For example, primary analysis include adjustment by parasitaemia, which is very much dependent on the microscopy quantification methodology (considered as a study metadata). How differences in the quantification can affect the analysis, and how they can be discarded or controlled for in the analysis? Similarly, the methodology to determine kelch13 mutations can have different performance and drive to different minor allele frequencies: Is this relevant? If so, how this will be taken into account? A better description on which methodological variabilities are important and how to treat them in the analysis would be very helpful.

Response: Microscopy-derived parasitaemia is influenced by variations in quantification methods across studies (e.g., assumed WBC count per µL, number of fields read, reporting thresholds), which are documented as part of the study-level metadata. In the primary analysis, we will incorporate baseline parasitaemia as a covariate. Where applicable, we will adjust for the microscopy method (e.g., thick vs. thin film, WBC count assumptions) by including these variables through study-level stratification or random-effects meta-regression approaches. Sensitivity analyses excluding studies with missing or unreliable microscopy metadata will also be performed to evaluate the robustness of the findings.

For Kelch13 genotyping, we will document the genotyping methods used, utilising the genotype assigned and reported by the original authors. The study inclusion period includes studies published over the past decade. Genotyping methodologies and platforms have undergone substantial changes during this period.

- Who and where will the analysis be conducted? Analytical capacities (especially this kind of complex IPD analysis) is usually concentrated in the Global North, while the data to be analysed is originated in the Global South. How the team is planning to contribute to mitigate these capacity imbalances for the mid/long-term aim of ensuring that the means and capacity to actively participate in the analysis (and ideally conduct those analysis independently) is available where the health problem lies (subsidiarity principle)? Some discussion on this would be very helpful to build a responsible data sharing environment.

Response: We thank the reviewer for bringing this issue to our attention and share their sentiments. This IPD meta-analysis is a collaborative effort between researchers in malaria-endemic regions and global partners with established data infrastructure and analytical expertise. The MARC SE-Africa consortium coordinates the project "Mitigating Antimalarial Resistance in Southern and Eastern Africa," an African-based initiative funded by EDCTP3. The first, senior, and numerous other authors are located in malaria-endemic countries, where their institutions are affiliated with national malaria programs, universities, and research centres in sub-Saharan Africa.

We have partnered with the Infectious Diseases Data Observatory (IDDO) and WWARN, based at the University of Oxford, to ensure compliance with the FAIR principles (Findable, Accessible, Interoperable, and Reusable) of data stewardship. These partners provide secure

data transfer protocols, quality-assured repositories, and standardized analytical frameworks required by the funder. Importantly, this collaboration builds on an established structure of the WWARN research group, which has previously established the K13 study group (see reference 8), and complements ongoing efforts to promote equitable global research partnerships. Their long-standing partnerships and relationships, built over the years, encourage data submission with the data owners, which is essential to ensuring the success of this collaborative effort. Moreover, all data contributors from malaria-endemic countries are co-authors in the publication resulting from this analysis.

To address the subsidiarity principle and promote local analysis ownership, we have integrated capacity-strengthening components throughout the project. These components include collaborative data curation, protocol development, investigation, and joint statistical analyses involving early-career researchers and technical experts from participating African institutions. Furthermore, we are committed to ensuring skills transfer through joint authorship and online webinars.

Reviewer: 2
Dr. Dawit Assefa, KNCV
Comments to the Author:
The document is scientifically rigorous and well-organized, demonstrating a strong commitment to statistical integrity and ethical considerations. The analysis is thorough, and the conclusions are well-supported by the data.

Response: We thank the reviewer for taking the time to help us improve our manuscript. We greatly appreciate your insight and guidance.

While the introduction provides relevant background information, a clearer statement of the study's public health significance would enhance its impact. Additionally, a concise summary of the current knowledge gaps and the unique contribution of this analysis would help contextualize its importance.

Response: We appreciate the reviewer's valuable suggestion. While the introduction included background on artemisinin resistance and Pfkelch13 *mutations, we agree that the public health significance and knowledge gaps can be stated more explicitly. We have added brief statements clarifying the importance of linking genotypic markers to treatment response to inform malaria treatment policies and drug-resistant malaria response strategies. We also emphasize the limitations of previous research and articulate how our IPD meta-analysis provides a unique opportunity to address these gaps using standardized, patient-level data from diverse geographic regions. Please see additions made to lines 135-141: "Understanding the relationship between* Pfkelch13 *mutations and parasite clearance is of paramount importance for informing WHO classification updates of their association with artemisinin (partial resistance). Prior studies have primarily relied on aggregate data and are often limited to a few geographic regions, making it difficult to assess the clinical implications of emerging mutations across diverse transmission settings, particularly moderate-to-high transmission settings. This analysis will also include an investigation of any association between Kelch 13 mutations and the ACT therapeutic efficacy, an endpoint of greater interest to national malaria programmes than parasite clearance alone. This IPD meta-analysis brings together*

harmonized, patient-level data from multiple malaria-endemic regions, enabling a pooled analysis to robustly quantify the effect of specific Pfkelch13 genotypes on artemisinin-based treatment response."

Some statistical methods, particularly the selection of thresholds for phenotypic resistance, could benefit from further justification to ensure clarity for general readers who may not be familiar with prior studies. Similarly, a brief explanation of the rationale for using a normal mixture model to determine parasite clearance thresholds would strengthen the methodological transparency.

Response: We have expanded the Methods section to include an additional explanation for choosing phenotypic resistance thresholds. Please see line 344-352 of the manuscript: The selection of thresholds for phenotypic resistance (for instance, Day 3 positivity >5% and a $PC_1/_2$ ≥5 hours), will be based on WHO guidelines and prior pooled analyses that identified these thresholds as indicative of delayed parasite clearance but will be re-evaluated in this analysis. A normal mixture model will be applied to the $PC_1/_2$ distribution to define subpopulations of fast- and slow-clearing parasites empirically. This statistical approach is commonly used when phenotypic data exhibit heterogeneity, allowing for a more robust estimation of cutoff points than arbitrary thresholds alone. Please see further details in reference 38: White LJ, Flegg JA, Phyo AP, Wiladpai-Ngern JH, Bethell D, Plowe C, et al. Defining the in vivo phenotype of artemisinin-resistant falciparum malaria: a modelling approach. PLoS medicine. 2015;12(4):e1001823. PMID: 25919029

Specific points for clarification:

Line 25: Define Pfkelch13 for clarity.

Response: We have defined the Pfkelch13 *term in greater detail. Please see lines 27 of this manuscript. "The systematic review and associated meta-analysis aim to determine the correlation between* Pfkelch13 *(alleles present in the* Kelch13 *gene region of the P. falciparum parasite genome) mutant genotypes and both parasite clearance and clinical and parasitological response to ACTs from a globally representative dataset pooling individual patient data (IPD) from eligible published and unpublished studies."*

Line 32: Provide a brief definition of PRISMA-IPD.

Response: We appreciate the reviewer's suggestion. We have revised the sentence to incorporate a brief explanation of the PRISMA-IPD guidelines in the methodology section to clarify however, due to the journal's requirements on Abstract word limitations, could not be added to the abstract. Please see lines 168-173.

Line 39: Clarify the abbreviations IDDO/WWARN.

Response: We thank the reviewer for their insight. These abbreviations will appear in the affiliations list. We attempted to add them to the abstract, but this addition expands the abstract beyond the journal's required word limits. However, we provided the full name in the section directly following the abstract section and in the main body of the manuscript.

Lines 71-72: ACTs has already been defined earlier; consider removing the redundant definition.

Response: Thank you for this keen observation. According to journal and academic conventions, the abstract and main text are treated as standalone sections. Therefore, we have retained the definition of ACTs (artemisinin-based combination therapies) in both the abstract and the introduction to ensure clarity for readers accessing either section independently.

Line 90: Specify what BTB/POZ refers to.

Response: We have revised the text to clarify that BTB/POZ refers to "a conserved structural domain within the Pfkelch13 gene, modulating protein–protein interactions". This region is of particular interest as several mutations associated with artemisinin (partial) resistance are located within or adjacent to the BTB/POZ domain.
The use of BTB/POZ in its current form has remained unchanged to align with widely used terminologies. Please see the example below:

https://pmc.ncbi.nlm.nih.gov/articles/PMC8765297/

VERSION 2 - REVIEW

**Reviewer**	**1**
**Name**	**Mayor, Alfredo**
**Affiliation**	**Instituto de Salud Global Barcelona**
**Date**	**21-May-2025**
**COI**	

My comments have been fully addressed. I have no further comments.

**Reviewer**	**2**
**Name**	**Assefa, Dawit**
**Affiliation**	**KNCV**
**Date**	**01-Jun-2025**
**COI**	

No further comment.
